# Fathers’ experiences singing to their infants: an interpretative phenomenological analysis

**DOI:** 10.3389/fpsyg.2026.1922180

**Published:** 2026-09-30

**Authors:** Kara N. Caine

**Affiliations:** Division of Music Education and Music Therapy, University of Kansas, Lawrence, KS, United States

**Keywords:** fatherhood, father-infant attachment, father-infant bonding, infant-directed singing, music therapy, musical parenting, home musical environment

## Abstract

**Background:**

Infant-directed singing (ID singing) is a powerful phenomenon with well-established clinical applications for infants and parents. Existing research, however, focuses primarily on mothers, leaving a critical gap in the literature.

**Methods:**

This study explores fathers’ experiences singing with their infants, specifically examining how fathers engage in and perceive singing to their infant; the factors that influence fathers’ singing; and how, if at all, singing influences father-infant bonding. Semi-structured interviews with thirteen fathers were analyzed using Interpretative Phenomenological Analysis (IPA).

**Results:**

Fathers in this study sang intuitively, expressively, and responsively to their infants. They perceived singing as a predominantly positive experience that regulated their emotions, although they also described occasional challenges with singing. Fathers’ singing was influenced by their heritage, spouse, and fatherhood role. While fathers expressed some uncertainty regarding singing’s immediate influence on father-infant bonding, many hoped music could help them relate to their child in the future.

**Discussion:**

Though findings align with previous research on maternal ID singing, participants’ accounts highlighted distinct aspects of their ID singing experiences. In order to meet fathers’ unique needs and promote equitable family-centered care, interventionists must account for fathers’ experiences when designing music interventions. Implications for music interventions and recommendations for future father-infant musicality research are discussed.

## Introduction

Caregivers across cultures and time have used singing to interact with and soothe their infants (Trehub and Gudmundsdottir, 2019). The way caregivers sing to their infants, or infant-directed (ID) singing, has distinct musical characteristics that capture infant attention and regulate arousal (Sharman et al., 2023; Trainor et al., 1997). Although some musical characteristics of ID singing differ between cultures, cross-cultural features include higher pitch as well as lower intensity and reduced roughness, indicative of a soothing and clear sound (Hilton et al., 2022). While infants and caregivers interact musically in a variety of ways (Malloch and Trevarthen, 2009), ID singing is often categorized into two styles, lullabies and playsongs, that differ in function. Playsongs stimulate and capture infant attention, while lullabies are sung to soothe and calm the infant (Trainor et al., 1997). In middle-class Western cultures, playsongs are sung more commonly, while lullabies are used more often when caregivers hold or carry infants frequently, as in agrarian societies (Trehub and Gudmundsdottir, 2019; Trehub et al., 1997).

Notably, most ID singing research focuses on white, Western, middle-class mothers (Cavero et al., 2024; Ilari, 2018). Evidence from this population indicates that ID singing benefits infants, caregivers, and the dyad (Sharman et al., 2023). Singing has been shown to regulate parent arousal (Cirelli and Trehub, 2020; Creighton et al., 2013), resulting in emotionally attuned caregiving that promotes parent-infant bonding (McLean, 2016; Sharman et al., 2023). The reciprocal nature of ID singing, or the ability for parents to adapt their singing in the moment to meet the infant’s needs, has been suggested to support parental sensitivity, which has long-term implications for the parent-infant relationship (de l’ Etoile, 2006).

Within this group of Western mothers, interpretive studies indicate mothers perceive ID singing as a positive experience that supports the developing mother-infant relationship and maternal well-being. Mothers share that singing regulates their emotions, lowers stress, and increases feelings of confidence and empowerment (Creighton et al., 2013; Mackinlay and Baker, 2005; Pixley, 2015). Mothers also indicate that singing helps them feel validated as important caregivers to their infant (Creighton et al., 2013) and aids in the process of identifying as a mother (Pixley, 2015). Brazilian mothers have shared similar musical parenting beliefs and perceptions as Western middle-class mothers, including that singing supports the transition to motherhood (Ilari et al., 2011). Despite the interpretive and empirical evidence that espouse the benefits of ID singing, there is a significant need to explore fathers’ unique experiences singing to their infant.

ID singing has been suggested as a component of interventions that support attachment-related outcomes, such as bonding, sensitivity, and maternal interaction for typically developing infants and parents. In a systematic review of music interventions to support the parent-infant relationship, interventions mediated by the child’s mother were rated as very effective and singing techniques in particular improved emotional co-regulation for healthy mothers and infants (Newman et al., 2022). However, more diverse participants and rigorous research designs are needed to determine the effectiveness of parental singing interventions. For at-risk populations, there is limited evidence that music interventions are helpful to support maternal wellbeing. One study found that mothers with moderate to severe symptoms of postnatal depression who attended a group music workshop that included singing had a faster reduction in symptoms when compared to participants in a structured play group (Fancourt and Perkins, 2018a). In the clinical setting, mothers in the Neonatal Intensive Care Unit (NICU) also benefit from engaging in singing experiences with their infants. One meta-analysis indicates that music therapy interventions, which included parental singing and other music therapy techniques, significantly decreased maternal anxiety (Bieleninik et al., 2016). Notably, fathers have been left out of music intervention research that investigates the parent-infant relationship and parental wellbeing.

When designing music interventions to support parents, understanding how fathers sing to their infants is critical as limited evidence indicates that fathers and mothers have similarities and differences when singing to infants. As with mothers, fathers alter their singing in the presence of their infants (O'Neill et al., 2001; Trehub et al., 1997). Adult listeners rate fathers’ ID singing as loving (O'Neill et al., 2001) and as emotionally engaging as mothers’ singing (Trehub et al., 1997). In one study, infants attended longer to fathers’ singing when compared to previous studies with mothers (O'Neill et al., 2001). However, fathers did not consistently raise the pitch of their ID singing in the laboratory setting. Significantly, raised pitch is associated with higher emotional expression, important for the development of the parent-infant bond (Trainor et al., 2000). Yet, when fathers were recorded at home, fathers and mothers adjusted their singing in similar ways by increasing the pitch and using slower tempos (Trehub et al., 1997). More research is needed to explore the acoustic features of fathers’ ID singing as well as infant responses to fathers’ singing.

Mothers and fathers describe similar caregiving contexts for infant-directed singing, including play, feeding, sleep routines, car travel, diaper changing and bathing. In one survey study, play was the highest reported context for singing and consequentially, playsongs were the most common song type used by parents (Trehub et al., 1997). Specific song choices are also linked to parent preference (Fancourt and Perkins, 2018b) and experiences with one’s own parents (Custodero, 2006; Gibson, 2009). Research suggests that fathers choose different songs to sing to their infants when compared to mothers. Fathers are more likely to sing or alter adult music or create songs for their infants while mothers are more likely to sing precomposed children’s songs (Gibson, 2009; Ilari et al., 2011; Trehub et al., 1997).

Studies that examine gender and singing have often explored the phenomenon of boys and adolescents leaving choral music education programs (i.e., “missing males”), with one possible explanation of singing being viewed feminine (Freer, 2010). As a response to this problem, all-male choirs have used hegemonic ideas of masculinity to recruit boys, reinforcing the gender binary (Palkki, 2024). Beyond childhood, studies of adult men and singing have focused on all-male choirs. Scholars suggest that singing in all-male choirs challenges traditional masculinity, and that singing is only considered feminine in certain contexts (Falconer, 2022; Harrison et al., 2012). In the caregiving context, singing to soothe an infant or care for children has been linked with femininity (Rolvsjord and Halstead, 2013), and lullaby singing in particular has been described as a “quintessential image of motherhood” (Pixley, 2015, p. 11). It is unclear how fathers understand masculinity and singing during caregiving, and if gender identity impacts how fathers interact musically with their infants.

Pooled data from the last decade suggests that fathers sing less than mothers and engage less in family musical experiences overall (Yan et al., 2021). Differences in singing may reflect differences in the time parents spend caregiving, as parental music engagement has been linked to engagement in non-musical activities (Steinberg et al., 2021). Notably, Western fathers in heterosexual marriages have significantly increased their involvement in childcare over recent decades (Bakermans-Kranenburg et al., 2019), with an unknown impact on fathers’ singing.

As Western fathers become more involved in caregiving, understanding how this group of fathers engage in musical caregiving, particularly singing, is needed. Fathers in this population may benefit from increased opportunities to sing to their infant not only due to the well-established benefits for parents and infants, but also because fathers have reported feeling left out of musical caregiving experiences such as early childhood music classes (Sanfilippo et al., 2026). Importantly, including fathers in research and intervention design is critical to develop interventions that are more inclusive for all families.

To address this gap in the literature and explore fathers’ experiences singing to their infants, this study will address the following research questions with a small group of fathers from the Midwestern region of the United States:

1. How do participants engage in singing experiences with their infants?
2. How do participants perceive the experience of singing to their infant?
3. What influences participants’ singing to their infant?
4. How, if it all, does singing to their infant influence father-infant bonding?

## Materials and methods

### Design

This study used interpretative phenomenological analysis (IPA), a qualitative approach that examines how people understand and make meaning of an experience (Smith, 2011). IPA has three theoretical foundations: phenomenology, hermeneutics and idiography (Smith et al., 2009). Phenomenology as a broad philosophical movement focuses on lived experience and hermeneutics involves interpretation to foster understanding (Eatough and Smith, 2017). IPA engages in a double hermeneutic, whereby the researcher interprets the participant’s experience, based on the participant’s own understanding of their experiences (Smith et al., 2009). Finally, IPA is idiographic, meaning that interpretations must stay true to the individual experience before comparing across cases (Smith et al., 2009). IPA was chosen for this study because it is especially effective for exploring complex and emotional experiences such as caring for a loved one (Smith, 2011; Smith and Osborn, 2015).

### Study procedures and participants

Recruitment procedures, materials, and informed consent were approved in advance by the university institutional review board (IRB ID: STUDY00143112). Fathers were recruited from community-based parent-infant classes (i.e., baby story time, music story time, new father classes) and parenting groups (i.e., breastfeeding support group, fatherhood group) within a mid-sized urban Midwestern city that houses a large public university. The researcher contacted local early childhood professionals (i.e., music therapist, early childhood music teacher) and a local fatherhood group who passed on information to their contacts and posted study information on social media. Flyers were also distributed at local businesses, the public library, and on the campus of the university.

Interested fathers contacted the researcher through the email address provided on the flyer. After confirming that the participant met inclusion criteria, an interview was scheduled at the participant’s preference, either the public library or in a private location at the university. After completing informed consent and a short demographic survey, fathers were interviewed individually by the researcher. The researcher used open-ended questions following an interview guide and all interviews were audio recorded. The interview guide was informed by previous research focused on the subjective experience of singing to infants (see Creighton et al., 2013; Custodero and Johnson-Green, 2003; Pixley, 2015; Shoemark and Arnup, 2014) and refined after a pilot interview and the first three interviews. The revised interview guide (see Appendix) added an additional question to allow for fathers to describe singing experiences that may not have been positive. The average length of the interviews was 28 min. Interviews were completed between February and April of 2018.

A purposive sample of 13 self-identified fathers of infants (18 months of age or younger) participated in this study. Although there is no prescriptive sample size for an IPA study (Smith and Nizza, 2022), a sample size greater than 10 could potentially challenge the idiographic nature of IPA. A relatively larger sample for this study allowed for data saturation and nuanced differences to emerge as only a single interview was completed for each participant, and 9 of the 13 interviews were brief (i.e., less than 30 min).

Participants were between the ages of 27–36, with an average age of 32. All fathers were the biological father, identified as male, were married, and lived full-time with the infant. Twelve fathers identified as heterosexual and one father chose not to identify his sexuality. Only two fathers had an older child in addition to their infant, and the remaining 11 of the 13 participants were first time fathers. Infant ages ranged between 7-weeks and 18-months old, however, the majority of infants (11 of 13 families) were between 5 and 10 months old. Fathers were primarily highly educated, middle class, and white. Detailed demographic information can be found in Table 1.

**Table 1:** Participant demographic information.

Pseudonym	Age	Ethnicity and/or Race	Highest level of education completed	Employment status	Yearly household income	Children
Arthur	31	White	Master’s degree	Employed full-time	$50,000–$74,999	7-month-old daughter
Beau	27	White, Cajun French	Master’s degree	Employed part-time, full-time student	$50,000–$74,999	7-8-month old daughter
Blake	27	White	Bachelor’s degree	Employed full-time	$50,000–$74,999	18-month-old twins (son and daughter)
Bob	31	White	Master’s degree	Full-time student	$50,000–$74,999	6-month-old son
Frank	35	White	Bachelor’s degree	Employed full-time	$20,000–$34,999	10-month-old daughter
George	33	European-American and ¼ Jew	Master’s degree	Employed full-time	$75,000–$99,999	7-week-old daughter; older daughter
Matthew	33	White, non-Hispanic	Doctoral degree	Employed full-time	$100,000–$149,999	9-month-old daughter
Michael	33	White	Bachelor’s degree	Stay-at-home parent	$75,000–$99,999	8-month-old daughter
Nathan	30	Caucasian/Latino	Master’s degree	Employed full-time	$75,000–$99,999	5-month-old son, older daughter
Russ	34	European American	Some college, no degree	Self-employed	$50,000–$74,999	7-month-old son
Ryan	33	Biracial	Master’s degree	Employed full-time	$50,000–$74,999	9-month-old son
Scott	36	White European	Master’s degree	Employed part-time	$100,000–$149,999	7-month-old daughter
Zack	32	White	High school degree or equivalent	Employed full-time	$20,000–$34,999	8-month-old son

Participants self-identified their race and/or ethnicity.

In addition to reporting demographic information, fathers were asked to describe their musical experiences and backgrounds. Six of the 13 fathers were professional or amateur musicians, and 10 participants mentioned past musical training. Regarding singing, six identified having formal (choir, musical theater, church, etc.) and/or informal singing experiences (making up songs, singing in the car), liking to sing, or having a good singing voice. Additionally, six participants sang openly in the interview when describing their experiences singing to their infant. Outside of music training, participants described music listening as the primary way they engaged in music daily. However, three participants shared that they rarely listened to music, and two of these fathers stated that the only time they were involved with music was with their infants.

### Analysis

Recordings were transcribed by an undergraduate research assistant and the primary investigator. Data were analyzed by the primary investigator in three broad phases: multiple readings and making exploratory comments; transforming exploratory notes into emergent themes; and seeking relationship and clustering themes (Pietkiewicz and Smith, 2014). A single investigator is suitable for the double hermeneutic in IPA, which relies on the researcher’s interpretation of the participants’ meaning making of an experience. To promote research reflexivity and credibility, the primary investigator kept a research journal and received feedback from research supervisors throughout the analysis.

In phase one, the primary investigator listened to all the recordings while reading each transcript. Each transcript was then re-read several times in order to actively engage with the data. Next, the primary investigator created exploratory comments that described the content of the interview, explored the language choices made by the participant, and questioned or interpreted the meaning of the data. In the second phase, exploratory comments were transformed into emergent themes at a higher level of abstraction.

In phase three, emergent themes were color-coded, compiled, and examined for recurrence across cases. Superordinate and nested subordinate themes were created using abstraction/subsumption and polarization. Themes were revised to ensure validity by reviewing the original transcripts. A narrative description of each theme was written, and a visual representation (table) of the themes was created. Participants were contacted via email after the initial interview for a member check and to give final approval for direct quotations.

### Researcher’s stance

I approached this study as a music therapist who has worked with typically developing infants, infants with disabilities, infants considered at risk, and infants in the neonatal intensive care unit (NICU). During the process of designing and conducting the study I became a first-time mother. My initial interest in researching fathers was in the clinical music therapy context of the NICU, but my interest soon grew to supporting all fathers and infants through music. I also have personal experience with “singing fathers.” First is my own dad, who sang the Beatles and Everly Brothers to me, and later, a husband who sang to our baby (a variety of songs and genres, but with a proclivity for Weezer and Bruce Springsteen). I am also a “singing mother,” and due to my professional and personal experiences, I planned some of what and how I would sing long before my baby’s arrival.

## Results

Fathers described how they sang to their infants, their perception of singing experiences, factors that influenced their singing, and the impact of singing experiences on bonding with their infant. Superordinate themes and nested subordinate themes are grouped by research question and outlined in Table 2.

**Table 2:** Superordinate and nested subordinate themes.

Research question	Superordinate themes	Subordinate themes
How do fathers engage in singing experiences with their infant?	Intuitively	Routines/rituals Songs fathers know Created songs
	Expressively	“Sometimes it’s soothing, more often it’s just silly” Performative
	Responsively	Infant regulation and engagement Alter musical characteristics
How do fathers perceive the experience of singing to their infant?	Regulates fathers’ emotions	Calm down Stay present Cope with serious medical concerns
	Some challenging experiences	When singing “fails” Public settings
What influences fathers’ singing with their infant?	Family history and culture	Competent parent Family songs
	Fatherhood role and identity	“Our thing” Separate from co-parent Shared with co-parent
How, if it all, does singing to their infant influence father-infant bonding?	Uncertain	Would have bonded other ways Singing as a way to be together
	Will be evident in the future	Creating memories Shared interest in music

### How do fathers engage in singing experiences with their infants?

Rather than describing singular significant events, fathers shared the myriad ways they sing to their infant during a typical day and reflected on how they have sung to their infant over time. Fathers sang intuitively, expressively, and responsively to their infant’s needs.

#### Intuitively

Fathers did not consciously plan when or what they were going to sing to their infant. Instead, singing was intuitive, spontaneous and natural. Fathers describe singing to their infants “all the time,” with several indicating that they sang everyday (Matthew, Michael, Scott, Zack). Two fathers, Bob and George, said they sang more than they talked to their babies. Although unplanned, singing was a part of the majority of fathers’ caregiving routines: during diaper changes, feeding, taking a walk, and/or bathing. Singing was also an important part of the nap or bedtime ritual, with fathers singing a certain song or sequence of songs each time. Blake, a music educator, describes that while music is highly valued in his family, he and his wife did not plan exactly which songs they would sing for his infant twins:

We have a sequence of songs that we just kind of stumbled into for bedtime. You know, starting a little more upbeat and getting slower as we go and softer as we go, that way it kind of lulls them to sleep [...] you’d think that we would’ve planned, being two musicians, that we would’ve thought about a different sequence or thought specifically, but it just kind of happened.

Fathers relied on their own musical repertoire in the moment and sang songs they already knew. Eight dads described how they sang across genres including bluegrass, country, folk, gospel, hymns, jazz/standards, rap, rock, and soul. Fathers sang these songs because they knew them by heart, or because they simply came to mind. Michael states “I just sing some goofy songs that I know. My mom was a huge Elvis Presley fan growing up so I know a gross amount of Elvis info and all his movies and songs by heart.” Even though he does not consciously plan what he will sing, Michael values these songs and the association to his mother.

Eleven of the 13 participants described creating songs for their infant, improvised in the moment, often about what he and the infant were doing. Nathan’s description is representative: “Sometimes I make up songs depending on the situation right now like, ‘Look at yourself, you’re in the mirror dancing,’ or something like that.” In addition to creating original melodies and lyrics, fathers also changed the lyrics to pre-composed songs. Several fathers shared that some of these made-up songs “stick” and are repeated by themselves and other family members. Bob states, “More often I sing tunes that I’ve heard before. But sometimes I’ll make them up and then I have found that if I do make them up, sometimes I’ll repeat them. So, I do have little tunes that I’ve made up that are now just songs for him.” Created songs become a part of the family repertoire. This shared music, like a family songbook, is unique to the family, and speaks to the intimacy and intuitiveness of singing.

#### Expressively

Fathers’ singing expressed different messages to their infants: “sometimes it’s soothing […] more often it’s just silly” (Zack). While fathers shared that they sang lullabies for their infant, fathers described singing playfully more often, describing play songs as “goofy” or “nonsense songs.” Fathers did not assign a purpose to these songs but noted that they were simply for fun. Arthur emphasizes his sense of humor when singing playfully to his daughter:

“I’ll kind of like joke-sing with her. My wife’s in the other room, I’ll sort of sing what she’s doing or I’ll jokingly be like, “Dad’s better than Mom,” or “Dad’s the best,” or something like that. Or I’ll kind of sing what we are doing. I just make up a lot of nonsensical songs […] It’s pretty funny, that stuff cracks me up.”

Further, fathers sang in exaggerated, “highly inflected” ways. George describes it as “the way that you would sing in a musical. When you have the love song and they’re singing loudly in each other’s faces.” George illustrates how singing to his daughter allows him to express his emotions in a performative way. Ryan also characterizes his singing as “theatrical” and “performative,” “like a standup comedian, seeing what his response is and what’s working for him and what’s not. And sometimes it clues me in if there’s anything else bothering him. So, he doesn’t speak yet, but that’s one of the ways that we can have communication about something that he can’t express quite yet.” This excerpt illustrates how singing functions as expression for both Ryan and his infant and shows how Ryan is responding to his infant’s cues.

#### Responsively

Like Ryan’s example, fathers sang in response to their infant’s needs, using singing as a way to regulate the infant’s affect. Ten fathers indicated that they sang to soothe their infant or to help the infant fall asleep. Similarly, six fathers described singing to distract the infant when fussy, or to occupy the infant’s attention. Scott describes the strategic way he uses singing with his infant: “And after you do bottle, comforting, diaper change, feeding, you’re starting to run out of ideas and it’s, ok, try singing, trying to get her back to reset to a ground state.” The majority of fathers perceived their infants as responding positively to their singing and shared that singing elicited the infant’s attention: “I think she’s just in the moment with me” (Frank).

Fathers also altered the characteristics of the music based on their infant’s response. Some fathers described how they would respond to their infant by imitating the sounds the baby was making. Three fathers shared how they might gradually slow the tempo and lower the volume of what they were singing to help soothe their infants. Due to the infant’s development, two musician-fathers recognized the need to keep the music simple. Russ expresses his surprise at his infant’s responses to children’s music: “I thought, ‘Oh, the nursery rhyme stuff, we’ll just skip that.’ But actually, she loves those songs and I think that’s fascinating that ‘Twinkle, Twinkle Little Star’ just is a song that babies love. Why do they love that?”

Fathers reflected on how their singing and their infant’s responses changed over time. The newborn period was unique, in that some fathers sang more composed songs, as opposed to improvising songs in the moment. For Blake, singing was the best way to soothe his twins. He describes how he and his wife sang more when they were newborns: “When they were younger, they cared less what we were singing. So, when we put them to bed our routine would be a little bit longer.” Alternatively, some fathers sang less to their newborns, due to the infant’s intense needs, and their limited ability to respond socially. Matthew expresses his confusion and disappointment that his daughter did not react to music the way he had anticipated:

Early on, especially when we were struggling with her with the acid reflux and the sleep issues, you know, week six and week seven, you want to say like, “See? Here’s the guitar,” and “See? Here’s music.” And like, “Settle down and be calm.” And there was a period of time where I thought like, “None of this is making any difference. None of this is taking at all,” but I just kept at it, and when she hit 3 months, 4 months, I really feel like she’s kind of making some of those connections.

Matthew later shared that he continued to sing to his infant despite her (lack of) response because he intuitively felt that music was good for her, in addition to being important to him. The majority of fathers in this study would agree with Matthew and shared the belief that music was beneficial for their infant.

### How do fathers perceive the experience of singing to their infant?

Fathers were overwhelmingly positive about their own experiences singing to their infant. Fathers described how singing could help them regulate their own emotions though fathers occasionally encountered challenges to their singing.

#### Regulates fathers’ emotions

Fathers described that singing helped them calm down, control negative emotions, and lower stress during frustrating moments. Several fathers mentioned how singing could be a positive feedback loop: “I think singing, it’ll calm myself and therefore I think that energy’s kind of transferring and it calms my daughter to get ready for nap time” (Michael).

Singing was a way for fathers to stay present and focus on the baby. Fathers could return to singing when they were preoccupied, to center themselves and keep their minds engaged. George states “I have to look at the kid if I’m singing to them. Which I mean it’s a less distracted state of parenting. If I’m singing to them, I am fully engaged with them.” In contrast, Scott described his experience as “nerve-wracking and fearful because I have a love-hate relationship with my voice.” Despite this, Scott described the same benefits from singing as the other participants, including that singing helped him stay calm and focused when trying to soothe his infant.

Beyond the daily stressors of parenthood, singing helped two fathers manage their infant’s serious and unexpected health concerns. Russ describes how he was able to use music to help his family through his daughter’s daily treatments for cystic fibrosis:

[Music] was something that kept track of the time without me checking the clock all the time, that helped me to relax and again, gave me something to focus on, some songs I might sing along with her and it went from being something that we all hated to just no big deal or even sometimes fun. I think that’s probably my biggest achievement in using music to turn the situation around.

Bob describes his experience singing to his infant in the neonatal intensive care unit (NICU):

It was really important in the NICU to sing him songs. We felt, and my wife sang with me sometimes, not always, but I know she also felt it was important, that I sing to him, that that would be a good way for him to get to know our voices. I guess if there’s one thing I want to highlight, it’s that it was really important for us to do that and it helped us through that experience.

Both fathers used music to benefit their infants but found that it also helped them cope with the experience. Even though the infants’ medical needs were not universal for this group of participants, Russ and Bob’s experiences mirror how fathers employed singing more generally with their infant; fathers intended for singing to benefit the infant but discovered it helped them stay regulated as well.

#### Occasional challenges

Six fathers described moments when singing did not “work” or failed to achieve their desired outcome (e.g., soothing a fussy baby). Fathers explained that this was likely because their infant had urgent needs that singing could not meet: to be fed, to be changed, or to sleep. Ryan expresses this sentiment using metaphor: “Sometimes he’s so upset or so tired that his ears are just stopped up. That means, “Oh, he’s probably cold” or there’s something more immediate, more pressing, lower on that Maslow scale that I’m not taking care of.” When the baby does not respond in the way fathers were wanting, they felt disappointed, like they were doing everything wrong, or “like a failure.” Matthew stated, “There have been times that she’s been really sick, like little kids get, with an ear infection or high fever, and you want to placate them in some way and again, singing is kind of one tool and I’ve seen it fail, right? Because it gets overwhelmed by other things she’s feeling. And that feels not great.”

Fathers also described a reluctance to sing at times, including outside of the home. George states, “There’s not an internal tension of ‘I wanna sing,’ it’s like, there are inopportune moments.” Several fathers described how it could be inappropriate to sing in particular public places, such as the library, a restaurant, or store; perhaps these “inopportune moments” are more accurately described as inopportune settings. Blake describes how, despite feeling self-conscious, he sang to his infant twins in public regardless: **“**When they were tiny there was no other option. I mean they were crying and that’s how they respond- they respond the best to music, to singing. So, walking through the aisles of Target singing to the cart, I felt a little bit weird about it […] If [wife’s name] was doing it I wouldn’t think it was weird, or I don’t think anyone else would think it was weird.” Similarly, Ryan explains the intimacy of singing in private, domestic spaces in contrast to parenting in public:

I’m not the sort of person who would sing outside of the house […] and my dad was the same way, very different public face to the six people that he let into his private sphere. All very close family members. So, personally, being able to do that with [infant’s name] and with my wife is a very intimate, like creating a family space and filling it with that stupid song about cereal or whatever it’s about. So, it speaks really strongly of our relationship and domesticity. Because I would not usually find myself doing it in a public park or something.

Despite occasional challenges, fathers persisted in singing with their infant. Like Ryan, some fathers looked towards a role model, while others relied on their belief that singing was important.

### What influences fathers’ singing with their infants?

Several factors impact fathers’ singing with their infant. Fathers’ family history and culture influenced fathers’ view of singing as well as the songs fathers chose to sing. Singing became a part of fathers’ role within the family, which could influence or be influenced by their spouse.

#### Family history and culture

The majority of participants shared how one or both parents sang to them as children, in both playful and soothing ways. Some fathers recognized singing as a way to be a competent parent. Bob describes how seeing his parents sing to his younger sibling showed him “That’s how I’ve seen people parent, is they sing.” Matthew also stresses how singing is one aspect of being an involved father, which he saw modeled by his uncle, rather than his father: “I think, the singing and the reading - like one day a week I work from the house so that I can help out with her care, and just be there with her, and I see it all as trying to be really involved and connect with her.” Zack states that singing makes him “feel more like a dad. More emotionally present, I guess. Like, my dad never sang to me. I don’t know if that has anything to do with it. More playful and child-like.” The act of singing may have helped participants embody the nurturing, involved father they hoped to become, perhaps in contrast to their own father.

Some fathers passed on the songs their own parents sang to their children: “my parents have told me that ‘We sang this song to you and this one’ and a couple of them I sing to my kids, too” (Nathan). Others, like Russ, adapted the tradition: “What I remember my dad singing to me are songs in Hebrew […] hearing those sounds of a language that was unfamiliar but hearing him sing it very softly as I was going to sleep, I fell in love with the sounds of the language. And I don’t sing to her in Hebrew, but I do sing some of the Vedic chanting and I feel that’s in the same spirit.”

Two fathers identified how certain songs could connect their infant with important family members who had passed away. Michael shared how singing songs by Elvis is a way for his daughter to get to know his mother, who passed away after his daughter was born: “She won’t have any ties to her, essentially and if that one tie is Elvis, that’d be super cool to pass on.”

Beau connects the songs he sings to his daughter with his grandparents, particularly his maternal grandmother. In addition to being songs he knows very well, they have a connection to his cultural heritage:

I sing to her the soundtrack of “Oh Brother Where Art Thou.” My best friend growing up, like second or third grade, we would just listen to it all the time, and I think, that might have to do with my grandmother and the stories, and my grandfather and the stories he would tell us. My paternal grandfather and my maternal grandmother, they both grew up during the Great Depression, but as Cajun in Louisiana. […] I want her to know a bit of her heritage. My grandmother had passed 2 or 3 months before we found out that we were expecting. So, I guess I really wanted my grandmother to meet her in a way, or for her to meet my grandmother.

#### Fatherhood role and identity

Fathers described how singing was a part of their role within the family, creating special times with their infant during their caregiving responsibilities. George described the changing table as his opportunity to connect and sing with his infant in contrast to breastfeeding, which he is not involved in. Ryan illustrates how the songs he sings during his caregiving routines also become special, and that he, alone, elicits the strongest reaction from his son: **“**I hear that when other people sing, he’s not nearly as animated or engaged. So that nighttime lullaby seems to have become our thing. And I think I’m the only one that does the diaper changing song, at least with any regularity or consistency. So that’s become like our thing, and he’ll start cackling when I start doing it.” Having these special songs (“our thing”) with his son may allow Ryan to feel a sense of mastery and pride, helping to secure his identity as a capable father.

Although all fathers shared that their spouses sang to their baby, fathers were mixed about whether their wife’s singing influenced their own. Singing was often a separate experience for each parent. Fathers described differences in the type of music they sang, as well as how they sang to their infant. Michael describes having different set of songs than his wife: “She has her own stuff, and I have my own stuff.” George goes beyond repertoire to state that his wife sings to his infant, but “not to the same extent or the same way” as he does. Two fathers shared that they sing in a more playful, goofy way than their spouse. Matthew and his wife both have musical training; however, Matthew finds that his wife’s training as a singer and her personality influence her to sing in a less spontaneous and playful way:

I think she more frequently sings to her in an effort to soothe or calm and I do some of that, but I also do that a lot to play, so to kind of get her excited, wound her up, and have a good time. [My wife] is a quieter person and she also has a very nice singing voice, growing up in her community’s choir, and so I do not think she engages in the ad-lib sort of thing.

Fathers also described encouraging their partner to sing. Nathan shares how his wife has learned the songs he routinely sings at bedtime: “I usually am the one who does bedtime, but when I’m not home she does the bedtime routine and does those soothing songs at night. And also, she’s learned the Spanish songs I sing so she sometimes sings those as well.” Bedtime, and the singing that accompanies it, is typically Nathan’s responsibility as a parent, a part of his fatherhood role within the family.

Fathers also described how singing could be a shared experience with their co-parent. Fathers discussed how their partner taught or encouraged them to try new songs or showed them when or how to sing. Further, some fathers described times when both parents sang together. Frank shares that, “sometimes when [my wife]’s singing I’ll sing, and when I’m singing, she’ll sing […] Or I will sit there and I will think of a really good rhyme to go along with her.” Russ describes how singing was a nice way to be with his partner after becoming parents.

My wife and I aren’t getting to spend a lot of time together, we are hardly home and awake at the same time, so those moments where we can sing songs together- there are a couple of goofy parody songs that we made up in maybe the second trimester when things were just fun and mellow […] if I hear her singing it, I might join in and vice versa. So, the songs become the shared language of how we can be nurturing the baby.

Russ’s experience also reinforces how created songs can become part of the “family songbook,” and how this intimate, private way of caregiving can be one way to be a nurturing parent.

### How does singing to their infant influence father-infant bonding?

Many fathers expressed uncertainty regarding singing’s influence on bonding with their infant, yet fathers saw singing as a positive way to interact with their baby. Additionally, fathers looked to the future as when they would know if they were bonded with their baby.

#### Uncertain

The majority of fathers were unsure of the direct impact of singing on bonding, expressing that singing might play a role, but is not the only way to bond with their infant. Matthew states “I think it’s probably one tool in the arsenal [...] if I didn’t feel like I could do that, I would find other ways.” Fathers indicated that singing to their infant was something that they simply did. Bob stated “It never occurred to me I wasn’t going to sing to my baby, right? It was just always like, ‘Oh yeah, that’s a thing I’ll do.’ And, then I did it and I don’t think a whole lot about like, ‘Oh, am I connecting with him?’”

Although fathers did not deliberately choose singing as a way to facilitate bonding, several fathers describe singing as an enjoyable way to interact. Singing was something fathers did while he and the baby were “hanging out” (Arthur, Bob). Fathers described singing as a way to make a connection with the infant, or to create a shared space for Dad and baby; singing was one way to be together. The interaction between Dad and baby was of prime importance, perhaps more so than it was a singing interaction.

#### Will be evident in the future

Participants seemed to conceptualize bonding as something that would be apparent when the child was no longer an infant, rather than a process of forming a relationship with their infant now. Arthur states, “Um, well I don’t know. She’s so little you can’t really- I mean I think we’ve got a good bond. I think she likes being around me, she likes being around my wife. My wife sings to her a lot too, she sings a lot to her. Uh, I just think she likes the interaction, any interaction, […] it’ll be interesting when she gets older to see if that has an effect.” Instead, fathers explored how their child might be impacted by their singing in the future. Dads expressed a hope that their child would remember singing songs or making music with them. Nathan shares, “I hope when they listen to that song again, they remember me. That’s the main thing, that’s why I keep singing or dancing to the same songs so that when [my children], in the future, they recognize them and hopefully they’ll remember ‘Oh, yeah. I used to sing with or dance to this with my dad.’”

Several fathers also hoped to connect with their child in the future through a shared interest in music, whether listening to or making music together. These fathers expressed how highly they valued music, which they hoped to pass on to their child. Zack explains the connection of singing to his future bond with his son, starting with the powerful moment of meeting his son after his wife’s C-section:

I was the first one to hold him and just so nervous with that first baby. Like, are you sure you are just going to hand it to me? So, after you say like “hi” and stuff it just, [laughs] it’s so, I do not know. It just seemed like a natural thing to do because I want him to appreciate music as much as I do. And I want it to be a part of who he is or how we connect or bond with one another.

Although some fathers were not clear whether singing impacts bonding, fathers were hopeful for the impact music will have on their relationship with their child in the future and find that singing is an enjoyable way to be with the infant now.

## Discussion

Overall, the composite description of how these 13 fathers sing to their infants is consistent with previous ID singing literature. The findings indicate that, like mothers, fathers sing lullabies and play songs to soothe and engage their infants, and they alter their singing to meet the infant’s needs in the moment. Fathers describe singing playfully most often, which is typical for Western parents (Trehub et al., 1997) and two fathers shared that they sang more playfully than the infant’s mother. This observation aligns with prior research that indicates fathers engage in more challenging and energetic play in comparison to mothers (StGeorge and Freeman, 2017), as well as evidence that suggests fathers’ singing has different musical characteristics (O’Neill et al., 2001). It is possible that the participants’ propensity for using adult songs and improvisatory singing with infants, consistent with previous findings that indicate fathers are more likely to sing or adapt adult music (Gibson, 2009; Ilari et al., 2011; Trehub et al., 1997), is also characteristic of fathers’ distinct co-regulation style.

In contrast with previous research (Yan et al., 2021), many of the participants reported singing very often to their infants and, at times, more than the infant’s mother. This finding may reflect this small group of participants who self-identified as a ‘singing dads’ and chose to be interviewed for this study. Bob corroborates this: “One of the reasons I wanted to do this study is because I probably sing to him more than I talk to him, really […] I was like ‘I have to do this.’”

Although this group of fathers may not be representative of fathers overall, they exemplify a cultural shift in Western industrialized countries to a more involved fatherhood (Bakermans-Kranenburg et al., 2019). Several fathers in this study hinted at the limitations of a provider model of emotionally distant fatherhood and described ways in which they hoped to be more engaged and nurturing in comparison to their own fathers. In line with the evidence that singing helps mothers feel empowered and important, fathers in this study indicated singing was an avenue to being a capable father and a special way to be with their infant. Rather than being perceived as feminine, singing was a way fathers distinguished their caregiving role from that of their spouse. Further, some fathers in this study described influencing their wife’s singing, a unique observation that is not represented in extant research.

Perhaps the most surprising finding was that fathers were hesitant to connect singing with father-infant bonding. While previous research has focused on maternal bonding, the conceptual definitions of paternal and maternal bonding are similar. Bonding is an internal parent-driven process comprised of the emotions parents feel for their child (Bicking Kinsey and Hupcey, 2013; Nakić Radoš et al., 2024; Suzuki et al., 2022). Fathers in this study seemed to conceptualize bonding as something they would observe in the future, rather than their current emotional connection with their infant. Although extant literature is lacking comparing paternal and maternal bonding, evidence suggests that mothers and fathers have similarities and differences when comparing bonding trajectories over time (Unger et al., 2026).

Despite participants’ uncertainty regarding singing and bonding, participants described singing as a positive way to interact and connect with the infant, in line with research on paternal bonding and attachment (Condon et al., 2013; Suzuki et al., 2022). The connection between constructs of paternal attachment (Condon, 2015) and study findings, illustrated in Table 3, suggest that singing does have a positive impact on the father-infant relationship, regardless of whether fathers are aware of this effect.

**Table 3:** Alignment of study findings with paternal attachment constructs (Condon, 2015).

Attachment construct	Construct description	Relevant study findings
Patience and tolerance	Positive feelings and an absence of negative affect, such as boredom or irritability	Singing helped to avoid negative thoughts; fathers felt calm and in control of their emotions
Pleasure in interaction	Feelings of satisfaction and competence when interacting with the infant	Singing was enjoyable and elicited positive responses from the infant; singing is a way to be a good father
Affection and pride	Thoughts and feelings during infant interactions, such as ownership	Singing was special time spent with their baby, often separate from their spouse

### Implications for music intervention

Fathers were in consensus that singing was beneficial for their infant and reported singing as a positive experience for themselves as well. Like previous evidence with mothers (Creighton et al., 2013; Mackinlay and Baker, 2005; Pixley, 2015), participants described how singing could help regulate their emotions by keeping them calm and focused. Singing, therefore, is likely acceptable as a component of health promotive music interventions to support paternal wellbeing and father-infant interaction, despite the prevalence of interventions that primarily target mothers. Further, fathers may benefit from singing to cope with stressful caregiving situations, such as medical treatment or hospitalization of their infant. However, fathers may or may not recognize their own needs as important and, in particular, may not see father-infant bonding as an immediate need.

Parental emotional regulation, critical for a child’s social and emotional development, has been targeted for parenting and family interventions (Hajal and Paley, 2020). A parent’s emotional regulation is influenced by the unique emotional climate of parenting and may be especially challenging in the infancy stage (Hajal and Paley, 2020; Rutherford et al., 2015). Parental emotional regulation influences the quality of parenting behavior and impacts the entire family system, ultimately shaping the child’s social and emotional development (Hajal and Paley, 2020; Morris et al., 2007). Supporting fathers’ emotional regulation through a singing intervention, therefore, has implications for the entire family.

When designing music interventions for mothers and fathers, interventionists should understand fathers’ unique experiences and musical repertoire. Music therapists can use or adapt the technique of “song of kin,” a parent-chosen song, important to the family’s culture or one that has been passed on through generations (Loewy, 2013). In addition to precomposed songs, professionals should be aware that fathers may prefer to create or adapt their own music for their infant.

However, some fathers in this study expressed a reluctance to singing outside of the home, which may also extend to educational and clinical settings. Previous evidence indicates parents can have barriers to singing to their infant in clinical settings such as the NICU (Shoemark and Arnup, 2014). Music therapists working with fathers must build a strong therapeutic relationship to support fathers’ access of their musical agency, or the ability to use their musical resources in context (Epstein, 2024). There is no prescribed level of parental singing; instead, music therapists must collaborate with fathers and recognize they likely have different singing preferences than their spouse. Finally, therapists must also be aware of the impact for fathers if a singing experience “fails,” and offer support to navigate the potential effect on parental self-efficacy.

### Limitations and future research

The assertions made in this study reflect a small group of fathers and should be interpreted with caution. While this study was open to any self-identified fathers, the majority of participants were married, heterosexual, cisgendered, non-disabled, highly educated, middle class and white. At the same time, participants were varied in their music backgrounds and infants’ ages. There is the possibility of theoretical generalization for study findings, whereby the reader evaluates the transferability of this study’s results through their own lens of personal and professional experience (Smith et al., 2009). That being said, more research inclusive of fathers across cultures, genders, sexualities, relationship status, socioeconomic status and race is warranted.

This study reflects the experiences of fathers who self-identify as singing to their infants. Additional research is called for to investigate the experiences of fathers who do not typically sing, or who do not enjoy singing in general. One participant, Scott, did not find singing as a pleasurable experience, but sang to his infant because he believed that singing was beneficial for his infant’s development. Surprisingly, Scott described the same benefits of singing as other participants, including that singing supported his own emotional regulation.

Further research is needed to understand infant responses to fathers’ singing. Evidence from this study suggests that paternal singing elicits infant attention. However, O'Neill et al. (2001) found that infants did not differ in their attention between recordings of fathers’ ID singing and fathers’ infant-absent singing. Acoustic parameters of fathers’ live ID singing can also be analyzed to determine the similarities and differences between mothers’ ID singing. This information can contribute to the development of health promotive interventions for fathers and infants, as well as targeted interventions for infants and parents in clinical settings.

### Conclusion

This study makes a unique contribution to the literature by exploring fathers’ experiences of singing with their infants. Results are consistent with well-established ID singing research, while highlighting fathers’ unique experiences of how and why they sing with their infants in their everyday lives. Fathers’ perspectives must be taken into account in order for music therapists and music educators to meet fathers’ needs with potential impacts for the entire family. More information about infant behavioral responses to paternal singing as well as the acoustic features of paternal singing is needed to develop universal and targeted music interventions for father-infant bonding and attachment for clinical and nonclinical populations.

## Data Availability

The datasets presented in this article are not readily available because the participants of this study did not give written consent for their data to be shared publicly, and the interview transcripts cannot be fully de-identified to guarantee participant anonymity. Inquiries can be directed to the University of Kansas Human Research Protection Program/Institutional Review Board at irb@ku.edu.

## Author’s note

This project was completed in partial fulfillment of the doctoral degree at the University of Kansas. The author is now at the Department of Music Education and Music Therapy, The Ohio State University.
