# Supplementary material for: Fathers’ experiences singing to their infants: an interpretative phenomenological analysis
Source: Front Psychol. 2026 Sep 30;17:1922180. doi: 10.3389/fpsyg.2026.1922180 (PMC13661301; doi:10.3389/fpsyg.2026.1922180)
Supplement: Supplementary file 1 [file Supplementary_file_1.pdf]

Appendix

Interview Guide

1. In order to provide context for our discussion, tell me about how you use music in your daily life[1]

Potential follow-up questions: Did you/do you

- Learn or play an instrument[2]?
- Play in a band[3]?
- Participate in a school music ensemble (band, choir, orchestra, jazz band, etc.)?
- Sing in an ensemble (i.e. church choir) or for fun (i.e. karaoke)[2]?
- Take music lessons[2]?
- Attend live concerts?
- Listen to music?

2. Describe singing to your baby.

Potential follow up questions:

- When do you sing to your baby?
- What do you sing? (genre, song type or specific songs)
- How do you choose what to sing?
- What are the reasons for singing to your baby?

3. What do you notice about yourself when you sing to your baby? (thoughts, feelings, physical sensations, etc.)

4. What do you notice about your baby when you are singing[4]?

5. What effect, if any, does singing have on bonding with your baby?

6. How, if at all, does singing to your baby impact how you see yourself as a father?

7. What experiences do you remember or know of being sung to as a child?

- Who sang to you?
- What songs?

8. Describe an experience when your partner or another family member sang to your baby.

- Tell me if/how this changed how you sing to your baby.

9. When, if ever, have you felt like you can't sing to your baby?

- Describe those moments
- What holds you back in those moments[3]?
- What helps you work through those moments?

10. Do you use music with your baby other than singing (such as playing an instrument, listening to music, dancing to music, etc.)? If yes, please describe.

11. When, if ever, have you had an experience singing to your infant that was not positive?

---

[1] Pixley, M. M. (2015). *Singing motherhood: First time mothers' experiences singing to their infants* (Order No. 3720986) [Doctoral dissertation, City University of New York]. ProQuest Dissertations & Theses Global.

[2] Custodero, L.A. & Johnson-Green, E.A. (2003). Passing the cultural torch: Musical experience and musical parenting of infants. *Journal of Research in Music Education, 51*(2), 102-114. https://doi:10.2307/3345844

[3] Shoemark, H., & Arnup, S. (2014). A survey of how mothers think about and use voice with their hospitalized newborn infant. *Journal of Neonatal Nursing, 20*(3), 115-121. https://doi.org/10.1016/j.jnn.2013.09.007

[4] Creighton, A.L., Atherton, M., & Kitamura, C. (2013). Singing play songs and lullabies: Investigating subjective contributions to maternal attachment constructs. *Australian Journal of Music Therapy, 24*, 17-44.
